# First exploration of the on-treatment changes in tumor and organ uptake of a radiolabeled anti PD-L1 antibody during chemoradiotherapy in patients with non-small cell lung cancer using whole body PET

**DOI:** 10.1136/jitc-2023-007659

**Published:** 2024-02-01

**Authors:** Johanna E E Pouw, Sayed M S Hashemi, Marc C Huisman, Jessica E Wijngaarden, Maarten Slebe, Daniela E Oprea-Lager, Gerben J C Zwezerijnen, Danielle Vugts, Ezgi B Ulas, Tanja D de Gruijl, Teodora Radonic, Suresh Senan, C Willemien Menke-van der Houven van Oordt, Idris Bahce

**Affiliations:** 1 Department of Medical Oncology, Amsterdam UMC Locatie VUmc, Amsterdam, Netherlands; 2 Imaging and Biomarkers, Cancer Centre Amsterdam, Amsterdam, Netherlands; 3 Department of Pulmonary Medicine, Amsterdam UMC Locatie VUmc, Amsterdam, Netherlands; 4 Department of Radiology and Nuclear Medicine, Amsterdam UMC Locatie VUmc, Amsterdam, Netherlands; 5 Cancer Immunology, Amsterdam Institute for Infection and Immunity, Amsterdam, Netherlands; 6 Department of Pathology, Amsterdam UMC Locatie VUmc, Amsterdam, Netherlands; 7 Department of Radiation Oncology, Amsterdam UMC Locatie VUmc, Amsterdam, Netherlands; 8 Medical Oncology, Amsterdam UMC Locatie VUmc, Amsterdam, Netherlands

**Keywords:** Radiotherapy, Programmed Cell Death 1 Receptor, Non-Small Cell Lung Cancer, Tumor Microenvironment, Immune Checkpoint Inhibitors

## Abstract

**Background:**

In patients with locally advanced unresectable non-small cell lung cancer (NSCLC), durvalumab, an anti-programmed cell death ligand-1 (PD-L1) antibody, has shown improved overall survival when used as consolidation therapy following concurrent chemoradiotherapy (CRT). However, it is unclear whether CRT itself upregulates PD-L1 expression. Therefore, this study aimed to explore the changes in the uptake of the anti PD-L1 antibody [^89^Zr]Zr-durvalumab in tumors and healthy organs during CRT in patients with NSCLC.

**Methods:**

Patients with NSCLC scheduled to undergo CRT were scanned 7±1 days after administration of 37±1 MBq [^89^Zr]Zr-durvalumab at baseline, 1-week on-treatment and 1 week after finishing 6 weeks of CRT. First, [^89^Zr]Zr-durvalumab uptake was visually assessed in a low dose cohort with a mass dose of 2 mg durvalumab (0.13% of therapeutic dose) and subsequently, quantification was done in a high dose cohort with a mass dose of 22.5 mg durvalumab (1.5% of therapeutic dose). Tracer pharmacokinetics between injections were compared using venous blood samples drawn in the 22.5 mg cohort. Visual assessment included suspected lesion detectability. Positron emission tomography (PET) uptake in tumoral and healthy tissues was quantified using tumor to plasma ratio (TPR) and organ to plasma ratio, respectively.

**Results:**

In the 2 mg dose cohort, 88% of the 17 identified tumor lesions were positive at baseline, compared with 69% (9/13) for the 22.5 mg cohort. Although the absolute plasma concentrations between patients varied, the intrapatient variability was low. The ten quantitatively assessed lesions in the 22.5 mg cohort had a median TPR at baseline of 1.3 (IQR 0.7–1.5), on-treatment of 1.0 (IQR 0.7–1.4) and at the end of treatment of 0.7 (IQR 0.6–0.7). On-treatment, an increased uptake in bone marrow was seen in three out of five patients together with a decreased uptake in the spleen in four out of five patients.

**Conclusions:**

This study successfully imaged patients with NSCLC with [^89^Zr]Zr-durvalumab PET before and during CRT. Our data did not show any increase in [^89^Zr]Zr-durvalumab uptake in the tumor 1-week on-treatment and at the end of treatment. The changes observed in bone marrow and spleen may be due to an CRT-induced effect on immune cells.

**Trial registration number:**

EudraCT number: 2019-004284-51

**WHAT IS ALREADY KNOWN ON THIS TOPIC**

- Administration of chemotherapy and radiotherapy can increase the programmed cell death ligand-1 (PD-L1) expression on tumor cells as an immune evasion mechanism. In such cases, administering anti PD-L1 therapy may improve the overall treatment efficacy, which is the rationale for combining anti-PD-(L)1 agents with chemoradiotherapy in patients with locally advanced unresectable non-small cell lung cancer. Currently, multiple randomized clinical phase III studies are investigating this concept, including the PACIFIC-2 trial and ECOG-ACRIN EA5181 trials, where the addition of anti-PD-L1 durvalumab to concurrent chemoradiotherapy is investigated in this population.

**WHAT THIS STUDY ADDS**

- Our exploratory immuno-positron emission tomography (PET) findings with [^89^Zr]Zr-durvalumab suggest that in vivo anti PD-L1 tracer uptake changes rapidly (within 1 week) and systemically in response to chemoradiotherapy. Imaging after 1 week of treatment did not reveal the expected increase in tumor uptake of [^89^Zr]Zr-durvalumab. Instead, tumor uptake remained stable or decreased. In addition, spleen uptake tended to decrease, and bone marrow uptake to increase, likely reflecting leukocyte death followed by hematopoiesis and lymphocyte migration, respectively. Surprisingly, we observed the emergence of an immune-related symptomatic thyroiditis in one patient on PET-imaging at a dose as low as 1.5% of the therapeutic durvalumab dose, underscoring the profound impact of even very small doses of anti-PD-L1 therapy on the immune system. Both such biological effects as well as technical factors such as slow kinetics and the long-lived isotope raise concerns about the suitability of [^89^Zr]Zr-durvalumab PET for longitudinal assessment.

**HOW THIS STUDY MIGHT AFFECT RESEARCH, PRACTICE OR POLICY**

- Future immuno-PET studies investigating the changes in anti PD-L1 antibody uptake should use biologically inert tracers with fast kinetics and established quantification conditions to ensure accurate measurement, while minimizing any confounding effects on the immune system.

## Introduction

Programmed cell death ligand-1 (PD-L1) expression is a major defense mechanism used by cancer cells against immune-mediated cancer cell killing. In preclinical studies, the expression of tumor PD-L1 could be upregulated through chemotherapy-induced^1 2^ ^3^ and radiotherapy-induced^4^ immunogenic cell death. Both modalities cause the emergence of damage-associated molecular patterns (DAMPs), the release of cytokines (eg, type I interferons, interferon-γ), and the increased infiltration of pro-inflammatory immune cells (eg, dendritic cells, cytotoxic T lymphocytes), resulting in tumor PD-L1 upregulation.^5^

Platinum-based, definitive concurrent chemoradiotherapy (CRT) followed by consolidation anti-PD-L1 (durvalumab, 10 mg/kg every 2 weeks or 1500 mg every 4 weeks), is the guideline-recommended treatment in patients with locally advanced unresectable non-small cell lung cancer (NSCLC).^6^ A potential upregulation of tumor PD-L1 expression during chemotherapy and radiotherapy, supports the hypothesis that adding durvalumab from the start of CRT onwards could act synergistically with CRT. This approach is currently being investigated in the ECOG-ACRIN EA5181 trial (NCT04092283) and the PACIFIC-2 trial, both phase III, randomized, placebo-controlled studies (NCT03519971).

Assessing on-treatment tumor PD-L1 dynamics to understand whether PD-L1 expression becomes upregulated in patients with NSCLC is highly challenging, as this requires taking longitudinal biopsies. Biopsies are difficult to obtain and are limited in their ability to represent the entire tumor. Previous trials investigating matched tumor biopsies before and after neoadjuvant chemotherapy and/or radiotherapy showed varying levels of change in PD-L1 expression.^7–12^ However, these studies were limited by the long intervals between neoadjuvant treatment and tissues obtained at surgery, as well as the heterogeneity of tumor sampling.

These limitations can be overcome by positron emission tomography (PET) using repetitive “whole body PD-L1 imaging” scans to visualize early changes in the uptake of radioactively labeled anti PD-L1 antibodies, not only in one but in all tumor lesions at once, as well as in all non-malignant lymphoid organs and other tissues of interest. Different groups, including our own, have demonstrated before the safety and feasibility of [^89^Zr]Zr-N-succinyl-deferral-TFP ester (DFO)-durvalumab ([^89^Zr]Zr-durvalumab) PET.^13 14^

Therefore, the aim of the current study was to conduct an exploratory investigation into the changes in [^89^Zr]Zr-durvalumab uptake in both tumors and healthy organs during CRT in patients with NSCLC using whole-body immuno-PET imaging.

### Institutional review board

This monocenter PET imaging study was performed in accordance with the Declaration of Helsinki. The trial was registered at www.clinicaltrialsregister.eu.

### Patients

Patients with NSCLC, who were scheduled to undergo concurrent platinum-based doublet chemotherapy and thoracic radiotherapy (30 once-daily fractions of 2 Gy) were enrolled. For inclusion, patients were required to have at least one lesion with a diameter >1 cm. The main exclusion criteria were any prior therapies directed against PD-(L)1 and/or cytotoxic T-lymphocyte–associated antigen 4 (CTLA-4), active infection, autoimmune or inflammatory disease, or major surgical procedures within the past 28 days, as well as the use of immunosuppressive medication above physiological levels.

### Study design

**Figure 1:**
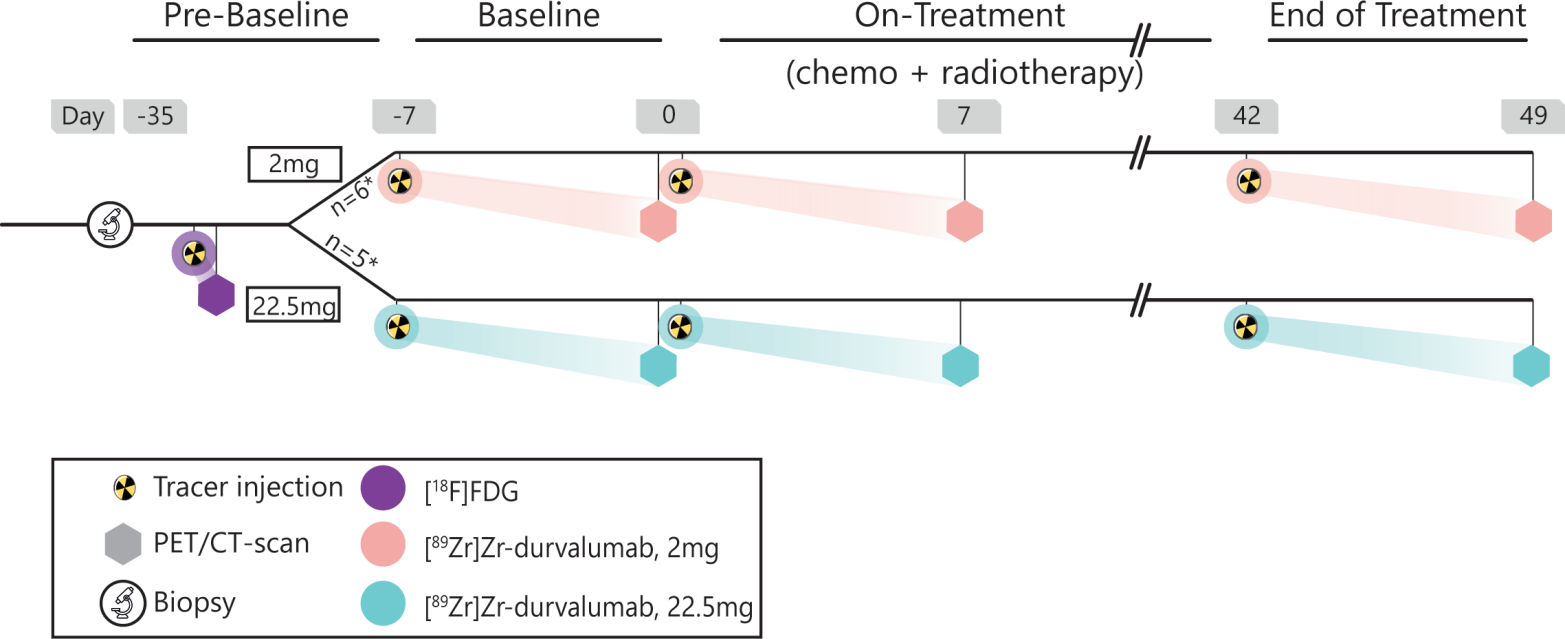
Graphical representation of the study design. Patients underwent a biopsy and a [^18^F]FDG PET/CT scan before baseline imaging. The first six patients entered the 2 mg cohort, the subsequent five patients the 22.5 mg dose cohort. Patients underwent three [^89^Zr]Zr-durvalumab administrations and subsequent PET/CT scans. The first chemotherapy administration and the first fraction of radiotherapy were planned on the day of the second [^89^Zr]Zr-durvalumab tracer injection. The last [^89^Zr]Zr-durvalumab administration was given at the end of the treatment (day 42), that is, the last radiotherapy fraction. [^18^F]FDG, [^18^F]F-fluorodeoxyglucose; PET, positron emission tomography.

The first group of patients was included in the 2 mg cohort, focused on optimal visual assessment. The second group in the 22.5 mg cohort, focused on reliable quantitative assessment (see below section “selection of mass dose levels”). All patients were scanned at baseline, 1-week on-treatment, and 1 week after finishing 6 weeks of CRT (end of treatment). Each PET-scan was performed 7±1 day after administration of 37±1 MBq [^89^Zr]Zr-durvalumab (online supplemental table 1), which is in line with the generally considered optimal time point in immuno-PET studies between 4 and 7 days post tracer injection.^15 16^ Compared with the two earlier reported [^89^Zr]Zr-durvalumab trials, which scanned at either 5 days or 3 and 5 days after tracer administration, our scans acquired at 7 days post administration will have reduced background signal at the expense of a slight reduction in the signal-to-noise ratio^13 14^. All patients underwent a clinical [^18^F]F-fluorodeoxyglucose ([^18^F]FDG) PET/CT scan for staging purposes prior to the baseline [^89^Zr]Zr-durvalumab imaging. The study procedures are shown in figure 1.

10.1136/jitc-2023-007659.supp1 Supplementary data

### Selection of mass dose levels

A different mass dose of unlabeled durvalumab was added to the tracer in the two dose cohorts, while the amount of radiolabeled durvalumab remained the same (~0.15 mg). For the low dose cohort, the mass dose was chosen to ensure best tumor visualization. This decision was based on the results of a recent [^89^Zr]Zr-durvalumab PET imaging trial in patients with NSCLC, where both 2 mg and 750 mg (therapeutic dose; 10 mg/kg/2 weeks) were used.^13^ For the 2 mg mass dose a higher lesion detection sensitivity was reported (25%) than for the 750 mg (14%). However, this very low dose had a suboptimal tumor supply as after 72 hours less than 5% residual plasma activity concentration remained in circulation compared with the injected activity (%IA/L).^13^ This makes quantification of the highly variable tumor uptake unreliable. Ideally, the optimal mass dose for accurate quantification approaches dose-independent clearance but does not saturate the tumor lesions. Clinical data from unlabeled durvalumab indicated that a minimum dose of 22.5 mg was needed to approach dose-independent plasma concentrations for up to 7 days after administration.^17^ Therefore, the dose level of 22.5 mg mass dose was chosen to administer in the high dose cohort.

### Immunohistochemistry and assessment of PD-L1 expression

Available baseline cytological or histological formalin-fixed paraffin-embedded tumor samples were collected to perform routine H&E and PD-L1 staining by immunohistochemistry (clone 22C3) on the Dako Autolitic stainer, which was validated against the PharmDx kit.^18 19^

An experienced pathologist assessed PD-L1 expression via the combination of tumor and mononuclear inflammatory immune cells, resulting in the combined positive score (CPS). The CPS is a better comparison to the total [^89^Zr]Zr-durvalumab tumor uptake than the Tumor Proportion Score (TPS), which is routinely used in clinical practice in NSCLC tumors and only includes PD-L1 expression on the tumor cells.^20^ CPS was demonstrated before to be equally predictive for immunotherapy efficacy, and in cases of TPS negative tumors even more predictive as compared with TPS.^20 21^ PD-L1 expression levels were divided into three categories: <1%, 1–49% and ≥50%.

### Tracer synthesis

[^89^Zr]Zr-durvalumab was produced in accordance with Good Manufacturing Practice at Amsterdam UMC, location VUmc, department of Radiology and Nuclear Medicine. Durvalumab is a fully human, immunoglobulin G1 kappa monoclonal antibody and was distributed by AstraZeneca, Cambridge, UK.^17^ Radiolabeling of durvalumab with ^89^Zr was done via the bifunctional chelator N-succinyl-DFO. The 2 mg [^89^Zr]Zr-durvalumab production in our center was previously described by Smit *et al*.^13^ For the preparation of the 22.5 mg [^89^Zr]Zr-durvalumab, the same procedure was followed; only more non-radiolabeled durvalumab was added in the formulation. Approximately 0.15 mg of the durvalumab was radiolabeled. The total tracer activity at calibration time was 37±1 MBq for all tracer productions.

### Scan acquisition and reconstruction

Whole body PET-scans were acquired on a clinical PET/CT-system, from head to mid-thigh in a feet first position, with 5 min per bed position acquisition time. The scans were reconstructed with time-of-flight iterative reconstruction into 4 mm^3^ voxel size. A low-dose CT-scan was performed at 120 kV, 30 mAs for attenuation correction and anatomical correlation.

### Pharmacokinetic analysis

In the 22.5 mg cohort, multiple blood samples were drawn after [^89^Zr]Zr-durvalumab administration, specifically at 15, 40, 70, 130 min and 48, 120 and 168 hours post injection. The radioactivity concentrations in these samples were measured in a well-counter in accordance with local procedures. The well-counter, dose-calibrator and PET-cameras were cross-calibrated. Tracer pharmacokinetics based on the measured radioactivity concentrations in blood and plasma were studied to compare tracer distribution and clearance between patients and time points.

### Image analysis

Visual assessment of the [^89^Zr]Zr-durvalumab PET-scans was performed by an experienced nuclear medicine physician. All lesions >1 cm that were confirmed malignant by pathology or considered highly suspicious based on the combination of increased uptake on screening [^18^F]FDG PET/CT-scan, radiological characteristics on CT and tumor drainage patterns, were visually assessed. In the 22.5 mg cohort, we quantitatively assessed up to three [^18^F]FDG PET/CT positive lesions per patient. This limit was applied to prevent data skewing by patients with multiple lesions; an effect what is especially disputable when effects vary significantly between patients but less so within the same patient. We selected the primary tumor in all patients, and when present, a metastatic mediastinal lymph node and a distant metastasis. For patients with multiple mediastinal lymph nodes, the node with the highest tumor to background ratio and clear anatomical demarcation was selected for analysis. The tumor lesions were delineated according to a previously published standard manual delineation method with the in-house developed tool for quantitative oncological molecular analysis: ACCURATE.^22 23^ Delineation was based on [^89^Zr]Zr-durvalumab uptake, with the low-dose CT as reference within ACCURATE. When tumor lesions were negative on the [^89^Zr]Zr-durvalumab PET scan, delineation was done based on the low-dose CT. Baseline [^18^F]FDG PET and diagnostic CT were used for anatomical reference for all patients. Non-suspicious lymph nodes in the head and neck region, the axillae/thoracic wall and the inguinal region that showed increased uptake above background on the [^89^Zr]Zr-durvalumab PET were counted.

Total tumor uptake was obtained as peak activity concentration to minimize the effect of interobserver variability and noise.^23^ It also limits uncertainties caused by PET/CT mismatch, since the highest uptake region is generally located in the center of the tumor. Standardized uptake value (SUV_peak_) normalizes the measured peak activity concentrations for body weight and injected activity; however, SUV_peak_ can only be used for comparison when tracer distribution and clearance is comparable between scans. To correct for differences in tracer supply, the tumor to plasma ratio (TPR) was calculated, based on the activity concentration measured directly before or after the PET-scan (168 hours). This measure can also be used for comparison with other patient populations and mass doses. Together with the TPR, the diameter of the lesion was evaluated and presented. In fact, a diameter smaller than 2 cm is known to cause size-dependent underestimation of the measured activity concentration and derivatives due to the low resolution of the [^89^Zr]Zr-PET scan (partial volume effect).^24^ Changes in tumor volume were based on the bidimensional axial axis on the low-dose CT, acquired directly before PET-acquisition.

Tracer uptake in healthy organs was measured as mean activity concentration and normalized for plasma activity concentration, resulting in an organ-to-plasma ratio (OPR). Uptake in the spleen, liver, bone marrow, brain, lungs, kidneys and thyroid was assessed by semi-automatic delineation of the complete organ with the in-house developed tool MVOI WB Dosimetry, either with or without predelineation by a convolutional neural network developed by Boellaard *et al*.^25 26^ Uptake in adipose tissue was automatically assessed by the corresponding CT Hounsfield units. For bone marrow and thyroid, the activity concentration was determined by the placement of one or multiple fixed size volume-of-interests in three adjacent slices. All delineations were based on the CT-slices and manually corrected in case of non-matching with the [^89^Zr]Zr-durvalumab PET uptake.

## Results

### Patients

**Figure 2:**
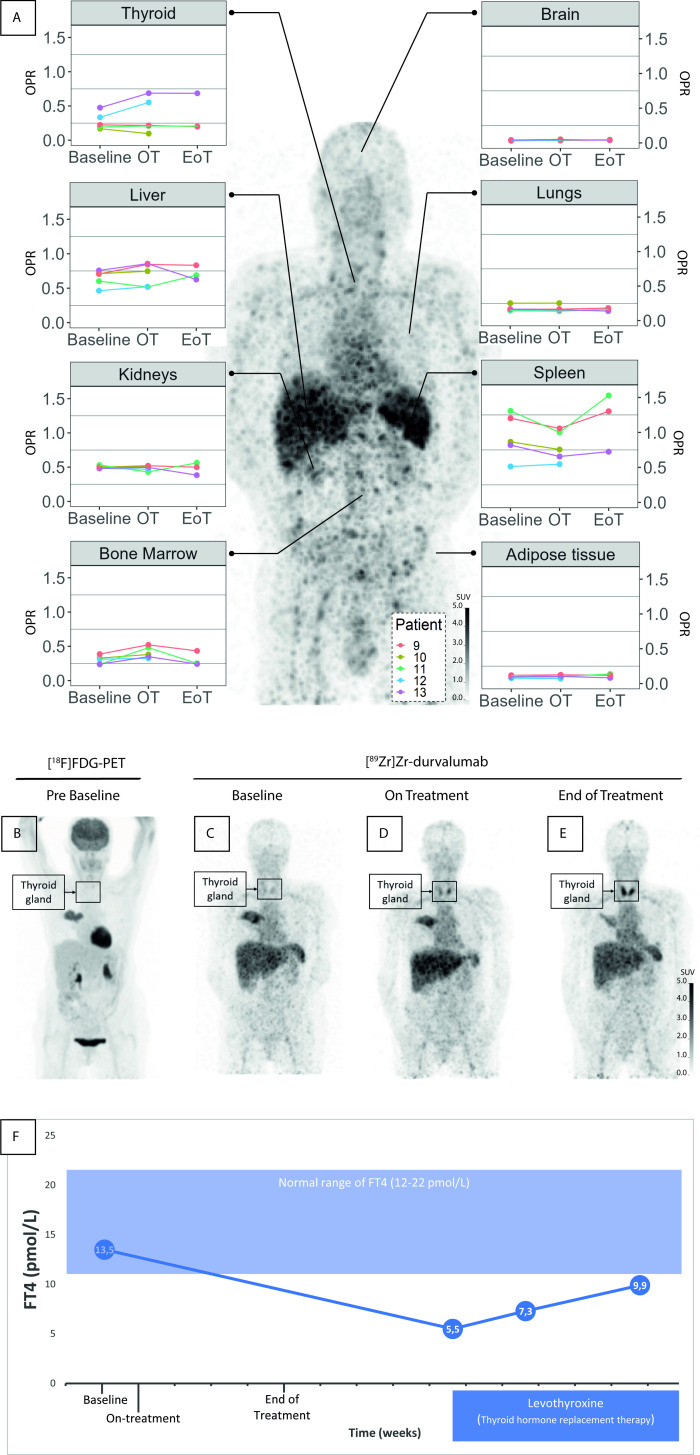
Healthy organ [^89^Zr]Zr-durvalumab uptake is shown for the 22.5 mg dose cohort, projected on the maximum intensity projection image of patient 8, at the end of treatment (A). On-treatment, the bone marrow showed an increase in OPR, while the spleen showed a decrease. Increasing OPR on-treatment was seen in the thyroid of a patient who had a known multinodular stroma at baseline (patient 12) and who developed hypothyroidism during treatment (patient 13) For this latter patient, the maximum intensity projection images of the [^18^F]FDG PET/CT scan (B) and the [^89^Zr]Zr-durvalumab PET/CT scans at baseline (C) on-treatment (D) and end of treatment (E) are shown. The gradual increase in [^89^Zr]Zr-durvalumab uptake in the thyroid can be observed. Per tracer injection, a mass dose of 22.5 mg durvalumab was given 1 week prior to each scan, amounting to a total of 67.5 mg in total over a period of 6 weeks. In the bottom row (F) blood analyses of patient 13 shows a decrease in FT4, indicating the occurrence of hypothyroidism under durvalumab, most likely due to an immune-related thyroiditis. The box in the bottom row indicates the start of thyroid hormone replacement therapy using levothyroxine with a subsequent increase in the FT4 levels. EoT, end of treatment; [^18^F]FDG, [^18^F]F-fluorodeoxyglucose; FT4,thyroxine; OPR,organ-to-plasma ratio; OT, on-treatment; PET, positron emission tomography.

A total of 11 patients with NSCLC underwent [^89^Zr]Zr-durvalumab PET imaging, of which 10 patients (5 in each cohort) had at least a PET-scan at baseline and one on-treatment. Additionally, 8 out of 10 patients also underwent the end of treatment scan. A flow chart of the patient inclusion is shown in online supplemental figure 1 and an overview of the patient and tumor characteristics can be found in table 1. In one patient (22.5 mg cohort), a grade 1 infusion-related reaction occurred (Common Terminology Criteria for Adverse Events V.5.0). In the same cohort, one patient who underwent all three [^89^Zr]Zr-durvalumab tracer administrations, developed hypothyroidism with thyroperoxidase antibodies, most likely due to an immune-related thyroiditis, see figure 2B–E.

**Table 1:** Patient and tumor characteristics of patients with at least one evaluable PET-scan (n=11)

Characteristic	Value
Age	65 (40–77)
Sex	n (%)
Male	6 (55)
Female	5 (45)
Histological subtype	n (%)
Squamous cell carcinoma	2 (18)
Non-squamous cell carcinoma	9 (82)
TPS at baseline*	n (%)
TPS<1	4 (36)
TPS 1–49	2 (18)
TPS≥50	4 (36)
TNM stage†	n (%)
IIIA	6 (55)
IVA	3 (27)
IVB	2 (18)
Previous anticancer treatment	n (%)
Stereotactic radiotherapy primary tumor	1 (9) (only 2/5 fractions)
Stereotactic radiotherapy brain metastasis	2 (18)
Bronchoscopic debulking primary tumor	1 (9)
ECOG performance score	n (%)
0	3 (27)
1	6 (55)
2	2 (18)
Smoking status	n (%)
Never (<100 cigarette in life time)	2 (18)
Former (stopped for >1 year)	4 (36)
Current (active or stopped for <1 year)	5 (45)
Total Gy during cCRT	n (%)
8‡	1 (9)
50	2 (18)
60	8 (72)
Type of chemotherapy during cCRT	n (%)
Carboplatin+pemetrexed	6 (55)
Carboplatin+etoposide	4 (36)
Carboplatin+paclitaxel	1 (9)
Progression-free survival§	Days (range)
Median	175 (5–not reached)

*One biopsy could not be evaluated, due to absence of tumor material.

†According TNM classification, UICC eighth edition.

‡This patient (7) died due to unknown cause after four fractions of radiotherapy. Only the baseline [^89^Zr]Zr-durvalumab PET-scan was performed.

§Progression-free survival from initiation of chemoradiotherapy until locoregional progression, occurrence of distant metastasis or death. Progression is not reached in one patient.

cCRT, concurrent chemoradiotherapy; ECOG, Eastern Cooperative Oncology Group; PET, positron emission tomography; TNM, tumor, node, metastasis; TPS, Tumor Proportion Score.

### Visual analysis—2 mg cohort

**Figure 3:**
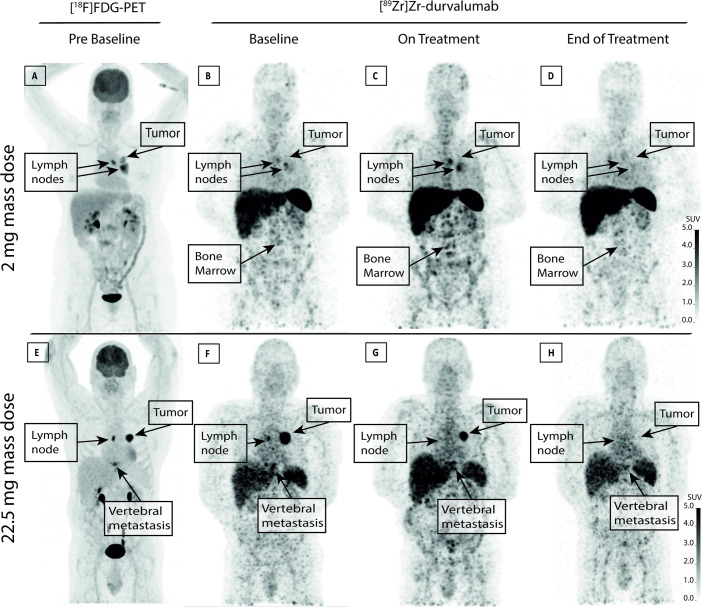
Maximum intensity projection images of a representative patient in the 2 mg cohort (A–D) and the 22.5 mg cohort (E–H) are shown. In the top row, the [^18^F]FDG PET scan of patient 2 (A) shows a tumor in the hilar region of the left lung with adjacent metastatic lymph nodes. The [^89^Zr]Zr-durvalumab scans at baseline (B) 1- week on-treatment (C) and after finishing CRT (D) are shown. The two metastatic lymph nodes show tracer accumulation at baseline. After 1 week of therapy, the uptake in these lesions appeared more enhanced, and the primary tumor could be detected. The tumor and lymph nodes could no longer be visualized after finishing CRT. Regarding the biodistribution, a higher uptake in bone marrow was observed after 1-week on-treatment compared with baseline, which diminished after finishing CRT. In the bottom row, for patient 9 in the 22.5 mg cohort, maximum intensity projection images of the [^18^F]FDG PET/CT scan show the primary tumor, the metastatic lymph node, and the vertebral metastasis (E). At baseline (F) high [^89^Zr]Zr-durvalumab uptake was seen in the malignant lesions. On-treatment (G) the tumor showed a substantial shrinkage of the tumor volume of [^89^Zr]Zr-durvalumab uptake, which corresponds with the tumor shrinkage measured on CT (figure 4A). At the end of treatment (H) hardly any [^89^Zr]Zr-durvalumab uptake could be seen in the primary tumor, vertebral metastasis, and lymph node. CRT. chemoradiotherapy; [18F]FDG, [^18^F]F-fluorodeoxyglucose; PET, positron emission tomography.

Out of the 17 [^18^F]FDG PET/CT positive lesions suspicious for malignancy, 15 (88%) were positive on the [^89^Zr]Zr-durvalumab PET at baseline and 14 out of 14 (100%, 3 lesions unevaluable due to a missing [^89^Zr]Zr-durvalumab PET-scan) on-treatment. Of these 14 lesions, 8 (57%) were still avid at the end of treatment [^89^Zr]Zr-durvalumab PET-scan. In all tumor lesions, the on-treatment tumor signal was either stable or visually enhanced compared with baseline for all patients, except for patient 5, in whom tumor uptake was decreased. At baseline, the highest uptake was seen in the liver, spleen, kidneys and bone marrow. Uptake was enhanced in liver and bone marrow on-treatment. At the end of treatment, tumor [^89^Zr]Zr-durvalumab decreased as well as the bone marrow uptake compared with the on-treatment scans (figure 3B–D). In all but one patients, non-suspicious lymph nodes could be visualized at baseline with increased [^89^Zr]Zr-durvalumab uptake (mean±SD per patient=7±6), with the highest number in the axillae and thoracic wall. In three patients, all lymph nodes became visually negative on-treatment, with partially but not complete reappearance on PET at the end of treatment (online supplemental figure 2). The other two patients had 7–40% less lymph nodes visible during/after treatment.

### Visual analysis—22.5 mg cohort

Regarding the tumors imaged, 9 out of 13 (69%) of the [^18^F]FDG PET/CT positive lesions, suspicious for malignancy, were also positive on the baseline [^89^Zr]Zr-durvalumab PET-scan. On-treatment, the percentage of positive [^89^Zr]Zr-durvalumab PET-lesions was the same (69%). Visually, no difference could be found in the on-treatment tumor signal compared with the baseline signal. At the end of the treatment, 55% of [^18^F]FDG PET/CT positive lesions were visible on the [^89^Zr]Zr-durvalumab PET-scan. Visually, the scans showed a similar pattern as the 2 mg cohort, with the highest uptake in the liver, spleen and bone marrow, and an increase in liver and bone marrow uptake on-treatment. In the 22.5 mg cohort, in some patients, non-suspicious lymph nodes were visible with increased [^89^Zr]Zr-durvalumab uptake, although on average less than observed in the 2 mg cohort (mean±SD per patient=2±2). In patients 9 and 11, respectively, 4 and 5 [^18^F]FDG PET/CT negative, non-suspicious lymph nodes were detected at baseline. During treatment, this number decreased (to, respectively, n=1 and n=2), and partially recovered at the end of treatment (n=4 for both patients) (see online supplemental figure 2).

### Quantitative analysis

To quantify changes in [^89^Zr]Zr-durvalumab uptake during treatment, patients in the high dose cohort were imaged with a mass dose of 22.5 mg. Additionally, venous blood samples were drawn to analyze intrapatient and interpatient variability of plasma tracer concentrations. An overview of the imaging data can be found in online supplemental table 1.

#### Pharmacokinetics

In online supplemental figure 3, the activity concentrations in plasma (A) and blood (B), normalized for injected activity (37±1 MBq), are visualized over time for the 22.5 mg dose cohort. The typical biphasic profile for antibodies can be distinguished, limited by the finite sampling time points, with a fast distribution phase (in the first 2 hours) and a slower elimination phase.^27^ There was a minimal intrapatient variability between the three tracer injections, but a substantial interpatient variability in plasma and whole blood activity concentrations. When comparing total tracer supply as a measure for clearance, the mean area under the time-activity concentration curve was slightly increased in four out of five patients on-treatment compared with baseline (online supplemental figure 3C), although not significantly (p=0.194, paired t-test). Due to missing data at the end of treatment, no formal comparison was performed for this time point.

#### Tumor [^89^Zr]Zr-durvalumab uptake related to clinical data

**Figure 4:**
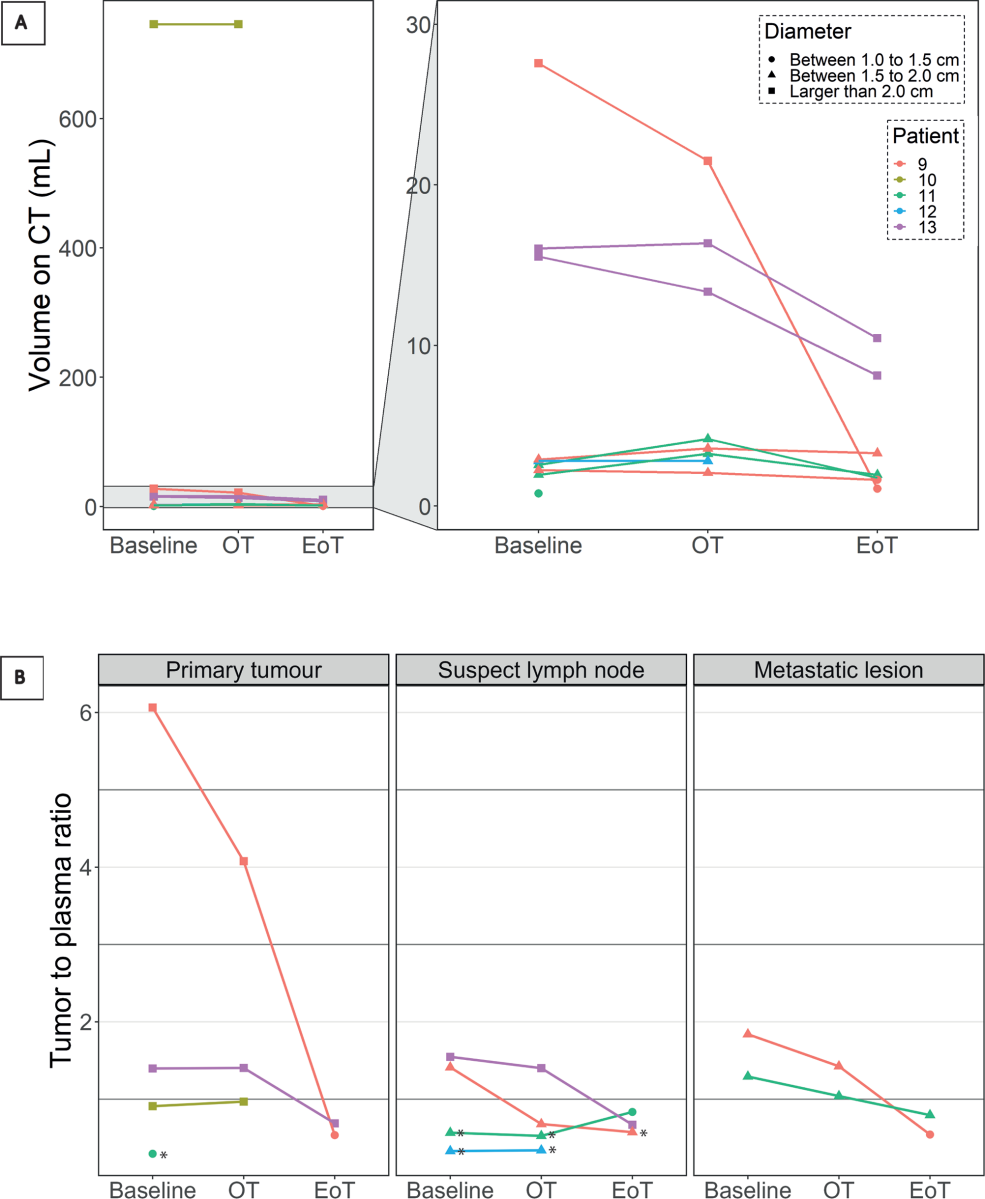
Changes in volume (A) and tumor [^89^Zr]Zr-durvalumab uptake (B) per lesion during concurrent chemoradiotherapy in the 22.5 mg cohort are shown. On-treatment, a large decrease in tumor volume (A) can be seen for the primary tumors of patient 9 and 13, while the volume of other lesions remained comparable. In the bottom row (B) a decrease in tracer uptake was seen on-treatment in several lesions: all tumoral lesions of patient 9, the metastatic lymph node of patient 13 and all metastatic lesions (patient 9 and 11). All other lesions retained a stable tracer uptake on-treatment. *Lesion that is negative for [^89^Zr]Zr-durvalumab uptake by visual assessment. EoT, end of treatment; OT, on-treatment; TPR, tumor to plasma ratio.

In total, four primary tumors, four suspect lymph nodes and two metastatic lesions were quantitatively assessed (see figure 4B). The tumors had a median (IQR) TPR at baseline of 1.3 (0.7–1.5), with the primary tumor of patient 9 showing extremely high uptake with a TPR of 6.1 at baseline. On-treatment, median (IQR) TPR was 1.0 (0.7–1.4). At the end of treatment, [^89^Zr]Zr-durvalumab uptake in the tumor had decreased for all lesions, except for the metastatic lymph node in patient 11. Notably, this lesion was not avid on baseline and on-treatment scans (noted with * in figure 4). The median (IQR) TPR at the end of treatment was 0.7 (0.6–0.7). The SUV_peak_ over time showed a similar pattern (online supplemental figure 4).

Patient 9, who had a high CPS (≥50%), showed a moderate to high tracer uptake in all tumoral lesions at baseline (TPR 6.1, 1.4, 1.8) and a moderate to large decrease in uptake after only 1 week of treatment, resulting in an on-treatment TPR of 4.1, 0.7, 1.4, respectively. The decrease in on-treatment TPR was also present, although in a lesser extent, in the oligometastatic bone lesion at the level of the 10th thoracic vertebra (third number), which was not included in the radiotherapy target volume. The primary tumor volume showed substantial regression (<−20%), although the metastatic lymph node increased in volume (>20%). Despite having an oligometastatic stage disease and having received 50 Gy instead of 60 Gy on the primary tumor, he achieved an excellent response to CRT with no recurrence of disease to date (ie, 19 months after finishing CRT).

Patient 10 (CPS of 1–49%) had a moderate tracer uptake in the tumor (TPR of 0.9). The tumor volume and TPR did not change on-treatment. Following CRT, the patient underwent a lobectomy with a partial chest wall resection, which showed a pathological complete response. This patient ultimately died from respiratory failure due to COVID-19.

In patient 11, the [^18^F]FDG PET/CT positive primary tumor did not show any [^89^Zr]Zr-durvalumab uptake before, during and at the end of treatment. The metastatic lymph node did not show any change in TPR on-treatment (TPR from 0.6 to 0.5) and was only avid at the end of treatment scan. The metastatic lesion in the sternum of this patient increased with 67% in volume between baseline scanning and the on-treatment scan, while [^89^Zr]Zr-durvalumab uptake decreased from a TPR of 1.3 to 1.0. This patient showed a relapse 14 months after finishing CRT.

The lesion of patient 12 was low on baseline [^89^Zr]Zr-durvalumab uptake (TPR of 0.3) and remained stable on-treatment in both uptake as well as in volume. In this patient, no relapse was seen to date (ie, 15 months).

Patient 13 (CPS 1–49), showed moderate uptake of [^89^Zr]Zr-durvalumab in the tumor (TPR of 1.4). The tumor volume and tumor TPR did not change on-treatment. Remarkably, this patient developed a thyroiditis, likely as an immune-related adverse event on the subtherapeutic tracer dose of [^89^Zr]Zr-durvalumab, while the tracer uptake in the thyroid increased on-treatment and even more at the end of treatment (see figure 2). Ultimately, thyroid hormone replacement therapy was required. After finishing CRT, consolidation of durvalumab was started, on which she developed a severe pneumonitis after one cycle, most probably due to the combination of previous radiotherapy and a therapeutic dose of durvalumab. Eventually, she recovered and is still free of disease to date (ie, 15 months).

#### [^89^Zr]Zr-durvalumab biodistribution

Regarding healthy organ uptake at baseline (see figure 2), high [^89^Zr]Zr-durvalumab organ uptake expressed in OPR was observed in the spleen (mean±SD=0.9±0.3), liver (mean±SD=0.6±0.1), kidneys (mean±SD=0.5±0.0) and bone marrow (mean±SD=0.3±0.1) with moderate and large interpatient variability in liver and spleen, respectively. Minimal to no activity was detected in adipose tissue and brain. In two patients with thyroid pathology, one of which was pre-existent, an increased thyroid [^89^Zr]Zr-durvalumab uptake expressed in OPR at baseline was observed (mean±SD=0.4±0.1), compared with patients without thyroid pathology (mean±SD=0.2±0.0). In these two patients, uptake increased during CRT (figure 2).

Remarkably, after just 1 week of CRT, an increase in bone marrow uptake was seen in three out of five patients simultaneously with a decrease in spleen uptake in four out of five patients. Patient 12, however, displayed an approximately stable uptake in bone marrow and spleen. At the end of treatment, the OPR in bone marrow had returned to baseline levels, but in spleen an increase above baseline was seen in two of the three patients at the end of treatment.

#### PD-L1 immunohistochemistry

Pretreatment samples from three primary tumors and two suspicious lymph nodes were analyzed for CPS on formalin-fixed paraffin-embedded sections, either freshly prepared or archival. CPS was not evaluable in only one case (patient 11), due to the loss of tissue architecture.

**Figure 5:**
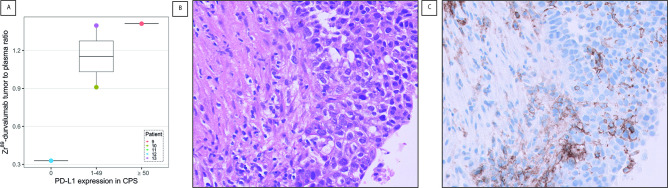
Tumor [^89^Zr]Zr-durvalumab uptake in tumor to plasma ratio (A) was low for patient 12 (CPS 0), intermediate for patient 10 (CPS 1–49) and high for patient 9 (CPS 1–49) and 13 (CPS≥50). The immunohistochemical H&E staining (B) is shown from the large tumor of patient 10 with ingrowth in the thoracic wall. PD-L1 staining of the tumor (C) with the 22C3 antibody was positive for PD-L1 at baseline with CPS of 10, containing many PD-L1 positive macrophages. CPS,combined positive score; PD-L1, programmed cell death ligand-1.

[^89^Zr]Zr-durvalumab uptake in TPR was intermediate to high (0.9–1.4) in the tumors that showed positive PD-L1 staining by CPS (figure 5).

## Discussion

To the best of our knowledge, this is the first clinical PET imaging study that longitudinally investigates the changes in uptake of an anti PD-L1 antibody during CRT in patients with NSCLC. The study shows changes in [^89^Zr]Zr-durvalumab uptake in both tumor and healthy organs within only 1 week after starting CRT.

### On-treatment changes in tumor uptake

**Figure 6:**
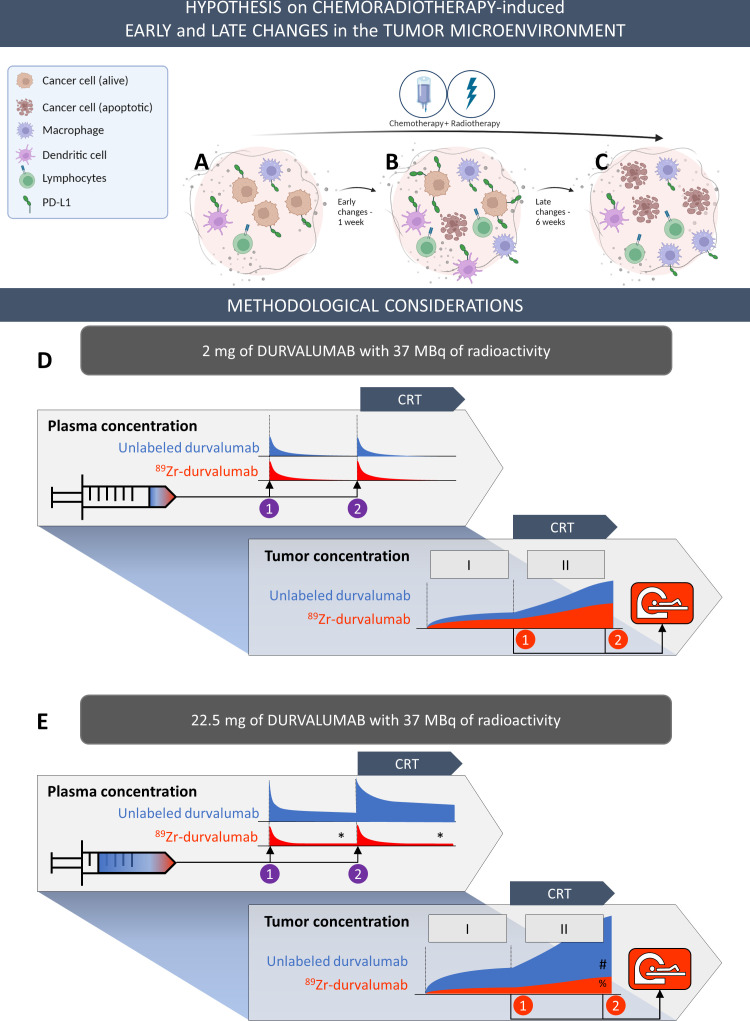
Schematic overview of different therapeutic (A–C) and methodological (D–E) effects that can influence the measurement of total tumor [^89^Zr]Zr-durvalumab uptake. Top row illustrates the hypothesis regarding the early and late CRT-induced changes in the TME. At baseline (A) PD-L1 can be expressed on tumor cells as well as immune cells (primarily on antigen-presenting cells). During treatment (B) we hypothesize that PD-L1 expression on tumor cells would increase in the early-phase, followed by the eventual death of PD-L1 expressing tumor cells due to the cytotoxic impact of chemoradiotherapy. PD-L1 expressing myeloid cells can migrate into or out of the tumor microenvironment (TME) to clean cell debris. These effects become more pronounced later in treatment (C). Since [^89^Zr]Zr-durvalumab binds to the PD-L1 receptor irrespective of cell type, the total uptake is influenced by these factors. In addition, the mass dose administered also affects tumor [^89^Zr]Zr-durvalumab uptake. Bottom rows represent the tracer features of the first two scans in the 2 mg and the 22.5 mg cohorts through a plasma and tumor tracer concentration profile. In both cohorts, durvalumab is administered twice at a 1-week interval (numbered purple circles) and scanned 1 week after each administration (numbered red circles). In the plasma and tumor profiles, the unlabeled durvalumab is shown in blue, and ^89^Zr-labeled durvalumab in red. Following the first tracer administration (1), the tracer binds the “baseline” tumor PD-L1 targets. Following the second tracer administration (2), the tracer binds to the hypothetically increasing tumor PD-L1 target concentration due to CRT. Regarding the methodological influences, in the 2 mg cohort (D) 2 mg durvalumab is administered, of which only a fraction (~0.15 mg) is labeled with ^89^Zr to a total activity of 37 MBq. With this low mass dose, dilution of the tumor signal due to unlabeled durvalumab is not expected. However, the reliability for quantifying uptake is limited because the tracer is rapidly cleared out of the blood compartment, leaving inadequate tracer concentrations to reach a reliable tumor to plasma ratio. This problem can be addressed by administering a higher mass dose (E). This approach has the advantage of having tracer available in circulation for a longer period (*), but with the risk of potentially diluting the tumor [^89^Zr]Zr-durvalumab uptake with much higher concentrations of unlabeled durvalumab in the tumor (#), thereby weakening the tumor signal (%). CRT, chemoradiotherapy; PD-L1, programmed cell death ligand-1.

We hypothesized that PD-L1 expression levels in the tumor would increase in the early phase of chemotherapy and radiotherapy as a defense mechanism, resulting in increased [^89^Zr]Zr-durvalumab uptake, with a decrease in the later phase due to cancer cell destruction. However, while in the 2 mg cohort a visual enhancement of the tumor [^89^Zr]Zr-durvalumab signal was seen 1-week on-treatment, this could not be quantified in the 22.5 mg cohort, where total tumor tracer uptake either stayed stable on-treatment or even decreased. The observed lack of increase in tracer uptake on treatment in the 22.5 mg dose cohort may be due to saturation effects, hindering detection of small receptor expression differences. Alternatively, it could also be due to the specific study design, which is further elaborated in the “Methodological considerations” section and visualized in figure 6.

### On-treatment changes in healthy organ uptake

Interestingly, on-treatment, a decrease in [^89^Zr]Zr-durvalumab uptake was seen in the spleen for the majority of the patients, while an increase was observed in the bone marrow (figure 2). The decrease in spleen uptake seemed to relate to the decreased number of non-suspicious lymph nodes that were avid on-treatment compared with baseline (online supplemental figure 2). This pattern could be explained by chemotherapy-induced leukocyte death, including leukocytes that express PD-L1. Additionally, leukocyte death can trigger compensatory hematopoiesis and the migration of B lymphocytes and effector T lymphocytes to the bone marrow, which could account for the observed increase in [^89^Zr]Zr-durvalumab uptake in the bone marrow.^28–30^

The baseline biodistribution of [^89^Zr]Zr-durvalumab is consistent with the PD-L1 RNA expression data from the human protein atlas.^31^ The high PD-L1 RNA expression reported in pulmonary macrophages may not have resulted in a high [^89^Zr]Zr-durvalumab uptake on PET in the lungs due to the limited number of cells in lung tissue compared with air. Although PD-L1 expression in brown adipose tissue is found in mice,^32^ the prevalence in the general population is low,^33^ just as the volume it compromises compared with all adipose tissue, matching the low amount of [^89^Zr]Zr-durvalumab uptake found in our patients. The order of baseline organ [^89^Zr]Zr-durvalumab uptake in this study is in line with previous reports of SUV data for [^89^Zr]Zr-atezolizumab^15^ and [^89^Zr]Zr-durvalumab (10 mg)^14^. However, the 2 mg [^89^Zr]Zr-durvalumab data from Smit *et al* differed in terms of their relatively lower kidney uptake.^13^

### Limiting study design characteristics

Although a sample size ranging from 10 to 20 patients is not uncommon for exploratory trials in the field of immuno-PET,^13 15 34–37^the total inclusion of 11 patients in this trial limits the extrapolation of the results outside the studied patient population. Furthermore, on the collection of study data, it became apparent that the current study design had other inherent characteristics that made it challenging to obtain representative outcomes to answer the research question reliably. These characteristics include both biological effects and methodological considerations, as elaborated below and visualized in figure 6.

#### Biological effects of therapy

The effects of CRT on PD-L1 dynamics occur as a result of therapy-induced immunogenic cell death in multiple ways. Although mitotic cell death (eg, due to DNA damage) may take some time, (interphase) apoptotic cell death leads to immediate release of DAMPs,^38 39^ followed by rapid release of interferons and subsequent PD-L1 upregulation within approximately 24–48 hours. When primed effector T cells are already present in the tumor microenvironment, these will become activated following tumor cell death and release interferon-γ (within 24 hours), further enhancing PD-L1 expression on tumor and/or myeloid cells, which extends PD-L1 expression in the tumor microenvironment by at least several days. In addition, interferon-induced dendritic cell activation and cross-presentation may lead to the priming of new waves of T cells (in the draining lymph nodes), which may be mobilized to the tumor in 1–2 weeks. Due to the continuation of cell death and the accompanied influx of immune cells, (mainly macrophages), these processes are likely to be enduring (visualized simplified in figure 6a).

In view of the expected early DAMP release and subsequent T-cell activation, we chose to perform [^89^Zr]Zr-durvalumab PET imaging 1 week after initiating CRT, aiming to catch the early effects of immunomodulation, without being hampered by massive cell death.

Nevertheless, since these two processes happen simultaneously and are variable between patients, the optimal balance between them is hard to find. For instance, the considerable radiological response observed in patient 9 after only 1 week of treatment suggests that earlier imaging might have been more revealing. This observation and the notion that radiological responses tend to underestimate the pathological response^40^ is crucial for informing future studies that aim to track longitudinal changes in PD-L1 expression using immuno-PET scans. It should be considered, moreover, that the immune cell dynamics as discussed above all contribute to the total measured [^89^Zr]Zr-durvalumab uptake

Besides the effects on tumor and immune cell behavior, CRT can also affect other biological factors in the tumor microenvironment (eg, perfusion, permeability, interstitial fluid pressure, hypoxia). These factors are known to influence antibody uptake in tumors and therefore contribute in a more or lesser extent to the observed changes in tumor [^89^Zr]Zr-durvalumab uptake.^41 42^

#### Biological effects of the tracer

PET imaging uses tracers at small doses, which are far below the therapeutic dose range. However, even at these seemingly low doses, tracers can have a biological effect. This is demonstrated by the gradual appearance of a durvalumab-mediated thyroiditis in patient 13, which began after a single administration of 22.5 mg [^89^Zr]Zr-durvalumab, equivalent to 1.5% of the therapeutic dose (1500 mg/4 weeks), as seen in figure 2. These undesired biological effects may interfere with the immunological processes that are intended to be studied, limiting the usefulness of this tracer.

#### Methodological considerations

The main methodological considerations of the current design come from features associated with [^89^Zr]Zr-durvalumab, including its long half-life and the need for tracer availability in plasma at later time points for adequate quantification of tracer uptake.

The choice of the mass dose (ie, unlabeled antibody added to the tracer) is critical for tracer quantification. Low doses can lead to high lesion detection sensitivity, that is, high signal in the target tissues such as the tumor lesions and to low background signal. However, this low dose may not adequately supply the tumor, as the tracer is rapidly cleared from the circulation to the larger “sink” organs where a large concentration of targets bind the circulating antibodies. Quantification then becomes unreliable due to quick clearance of tracer from circulation. On the other hand, high mass doses result in dilution of the PET signal with unlabeled durvalumab. The optimal dose for each antibody tracer must be determined based on pharmacokinetic data of the antibody and subsequent testing of different mass doses.^14 34^ Previously reported results with [^89^Zr]-Zr durvalumab support an optimal mass dose between 10 mg and 50 mg.^13 14^ In this study, the percentage [^89^Zr]-Zr durvalumab PET avid tumor lesions and the number of non-suspicious lymph nodes avid at baseline was lower in the 22.5 mg cohort (respectively, 69% ^89^Zr positive lesions and 2±2 lymph nodes), compared with the 2 mg cohort (88% ^89^Zr positive lesions and 7±6 lymph nodes), suggesting a saturating effect of the higher mass dose.

To enable longitudinal monitoring, multiple tracer administrations are required. However, stacking effects of the radionuclide and the parent tracer molecule must be considered, depending on their respective half-lives. In this study, stacking effects of durvalumab and the ^89^Zr-radionuclide had to be accounted for during on-treatment imaging. Based on low dose unlabeled durvalumab pharmacokinetic data (with an estimated half-life of 4 days), approximately one-quarter of the mass dose is expected to be still circulating in the blood during the second administration.^17^ Therefore, dose-adaptation of the second dose would be advised to account for this effect. Additionally, ^89^Zr cleaved from durvalumab after PD-L1 receptor-mediated internalization of the complex will residualize in the cell, resulting in stacking of the radionuclide. Based on its relatively long half-life of 78.41 hours, it is estimated to lead to an overestimation of the PET signal up to 15% in TPR with 1-week interval between tracer administrations.

### Immunohistochemistry

In the four patients for which CPS data was available, higher [^89^Zr]Zr-durvalumab tumor uptake was found for the patients with a higher categorical CPS. This finding, although based on very limited data, fits the (non-significant (p=0.06)) correlation between CPS and median [^89^Zr]Zr-durvalumab tumor uptake found by Smit *et al* in 11 patients with NSCLC.^13^

### Future perspectives

Using [^89^Zr]Zr-durvalumab to assess short-term changes in tumor PD-L1 dynamics in a clinical design to study the on-treatment effects of CRT is subject to a few constraints as described previously. Ideally, to better address this question, another PD-L1 targeted PET tracer with biologically inert properties, fast kinetics, and an established optimized dose should be used. An example of such a tracer is the ^18^F-labeled adnectin [^18^F]BMS986192^35^, but also the development of single-domain antibody-based tracers hold much promise.^43 44^ The presence of an unknown number of viable tumor cells and immune cells, with or without PD-L1 expression adds an extra layer of complexity and additional methods to assess these cells separately are needed. In future studies, decomposition of the different components that make up the total tracer uptake may become possible through multitracer imaging.^45^ This could be achieved by using different radionuclides and imaging time points, for example, by adding a myeloid cell tracer targeting the mannose receptor.^46^ Multitracer PET imaging can also increase understanding of tumor characteristics, such as perfusion^47^ or hypoxia^48^. Finally, the emergence of long-axial field of view PET-cameras marks a significant advancement, enabling high-sensitivity imaging while scanning the entire body in one acquisition position, instead of requiring multiple bed positions.^49^ For ^89^Zr-PET imaging, the use of this “total body PET-scanner” holds many advantages. We anticipate a lower noise-induced bias in the SUV_peak_ measurements^50^, together with enhanced lesion detection sensitivity, shorter imaging time and/or reduction in radiation dose, which can be quite extensive for ^89^Zr-labeled antibodies. The precision in imaging lung lesions, as in our trial, could be improved by applying ultrafast acquisition motion correction techniques^51^. Moreover, the long-axial field of view gives the option to perform kinetic analysis of tumors and organs simultaneously within one single dynamic scan; potentially while eliminating the need for burdensome blood sampling, since the aorta is almost certainly in the field of view.

## Conclusion

This study has conducted longitudinal imaging of patients with NSCLC undergoing CRT using [^89^Zr]Zr-durvalumab PET. Whereas an upregulation of PD-L1 expression on CRT was hypothesized, our data did not demonstrate a corresponding increase in [^89^Zr]Zr-durvalumab uptake in the tumor at 1-week on-treatment nor at the end of treatment. This observation is limited by the small sample size and multiple effects caused by subsequent imaging with a circulating antibody with a long half-life. Among the five patients studied, an increase in [^89^Zr]Zr-durvalumab uptake in the bone marrow after 1 week of treatment was observed in three out of five patients, while uptake decreased in the spleen in four patients. If these findings are confirmed in larger trials, this may indicate a CRT induced effect on displacement or killing of PD-L1 positive immune cells in the spleen and an influx or production of PD-L1 positive immune cells in the bone marrow, as early as 1-week on therapy. The study also underscores both biological and technical challenges in investigating changes in [^89^Zr]Zr-durvalumab uptake (in tumor lesions) during CRT. Future studies should consider exploring the use of a therapeutically inert PET tracer with faster kinetics to enhance our understanding in this area.

## Data Availability

Data are available upon reasonable request. Supplemental data are available online. Additional data are available upon reasonable request.
