# Supplementary material for: First exploration of the on-treatment changes in tumor and organ uptake of a radiolabeled anti PD-L1 antibody during chemoradiotherapy in patients with non-small cell lung cancer using whole body PET
Source: J Immunother Cancer. 2024 Feb 1;12(2):e007659. doi: 10.1136/jitc-2023-007659 (PMC10836378; doi:10.1136/jitc-2023-007659)
Supplement: Supplementary data [file jitc-2023-007659supp001.pdf]

SUPPLEMENTAL DATA

Supplemental Table 1. *Imaging data table shows for each patient the provided mass dose, whether any blood samples were drawn and the number of days between injection of [^{89}Zr]Zr-durvalumab and the PET-scan.*

			Days between injection and scan		
**Patient ID**	**Mass dose (mg)**	**Blood samples**	**Baseline**	**On-treatment**	**Post-treatment**
**2**	**2**	**No**	7	7	7
**3**	**2**	**No**	7	7	7
**4**	**2**	**No**	7	8	7
**5**	**2**	**No**	7	7	7
**7**	**2**	**No**	7	N.A.	N.A.
**8**	**22.5**	**Yes**	7	7	7
**9**	**22.5**	**Yes**	7	7	7
**10**	**22.5**	**Yes**	7	7	N.A.
**11**	**22.5**	**Yes**	7	7	7
**12**	**22.5**	**Yes**	7	7	N.A.
**13**	**22.5**	**Yes**	7	7	6

Abbreviations: *N.A. = not available*

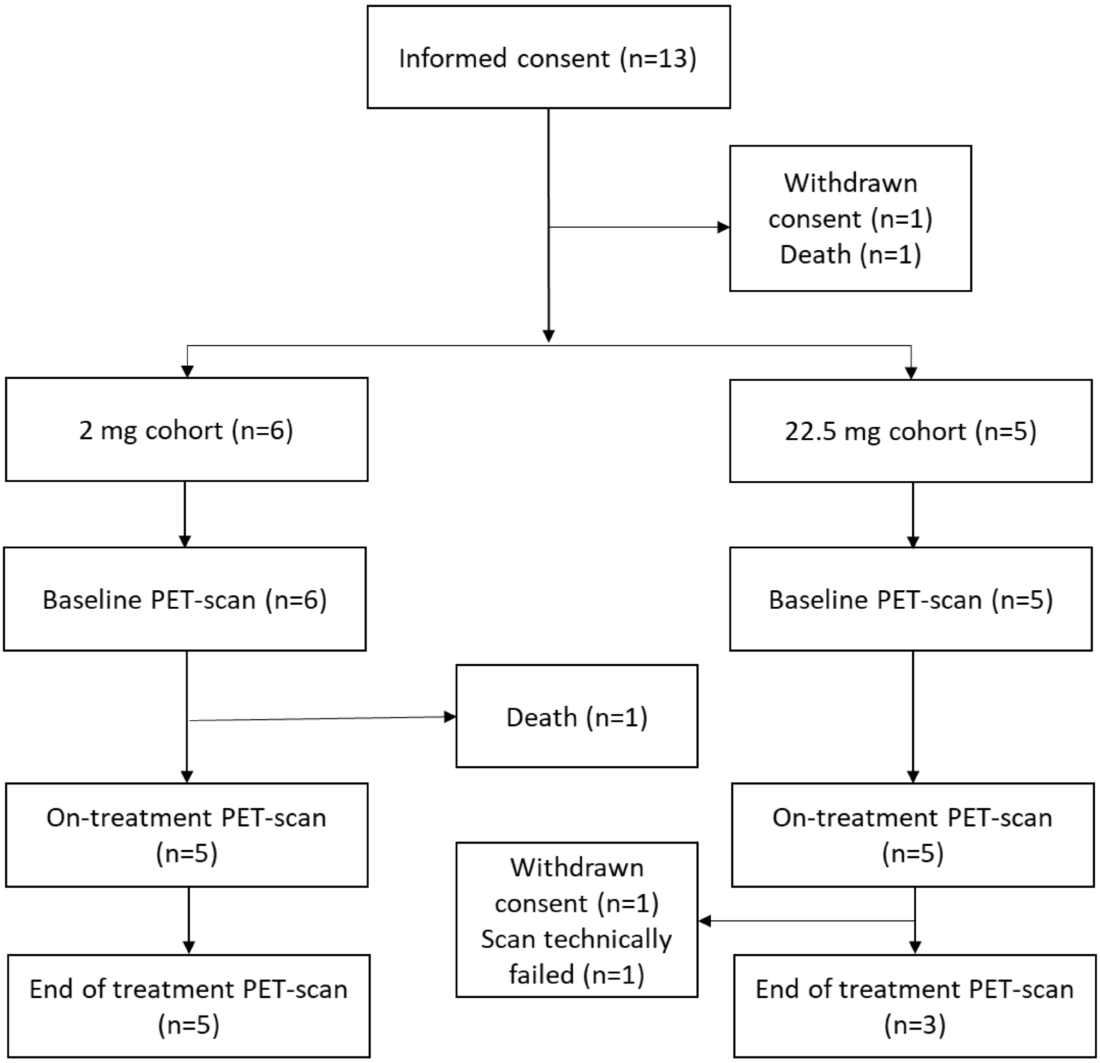

Supplemental Figure 1. *Consort flow diagram for the included patients. In total 13 patients were included, of which 11 underwent at least one [^{89}Zr]Zr-durvalumab PET – scan; 6 patients were scanned in the 2 mg cohort and 5 in the 22.5 mg cohort.*

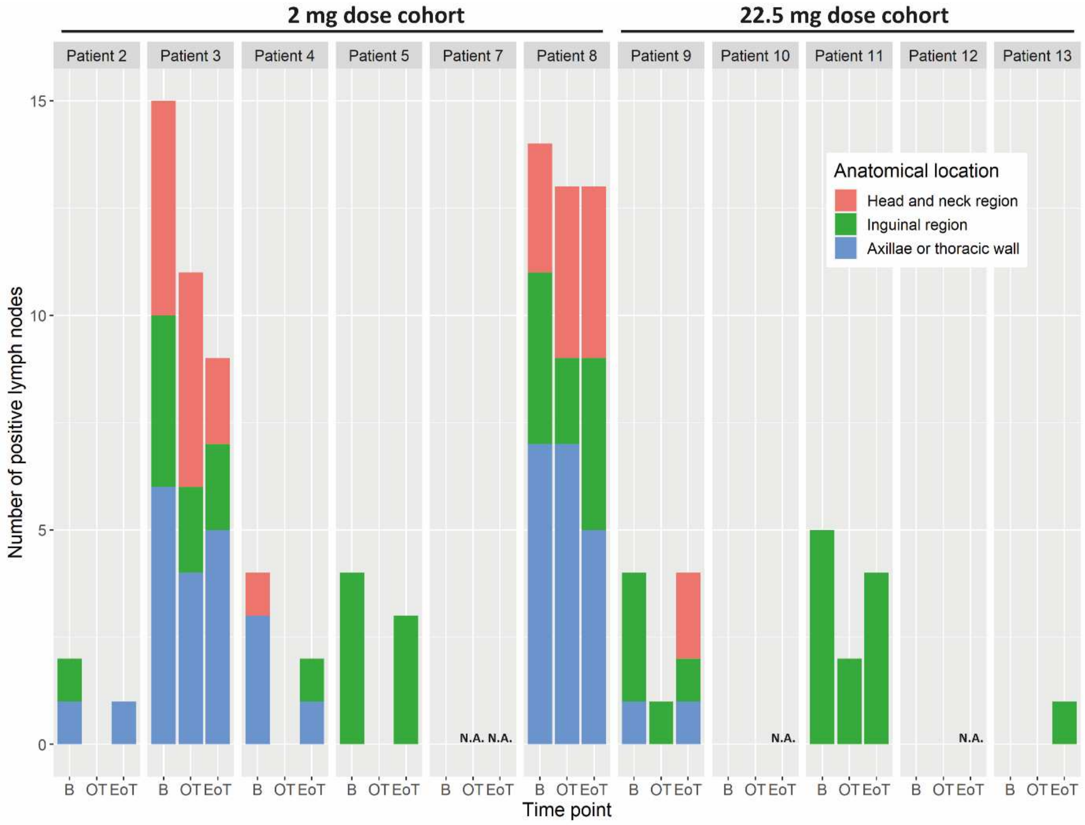

Supplemental Figure 2. *The number of [89Zr]Zr-durvalumab PET positive non-suspicious lymph nodes per patient in the head and neck region, axillae or thoracic wall, and inguinal region was higher for the 2 mg cohort (patient 2-8) than for the 22.5 mg cohort (patient 9-13) and decreased on-treatment for all patients with positive lymph nodes at baseline.*

Abbreviations: *B = Baseline, OT = On-treatment, EoT = End of Treatment, N.A. = scan not available*

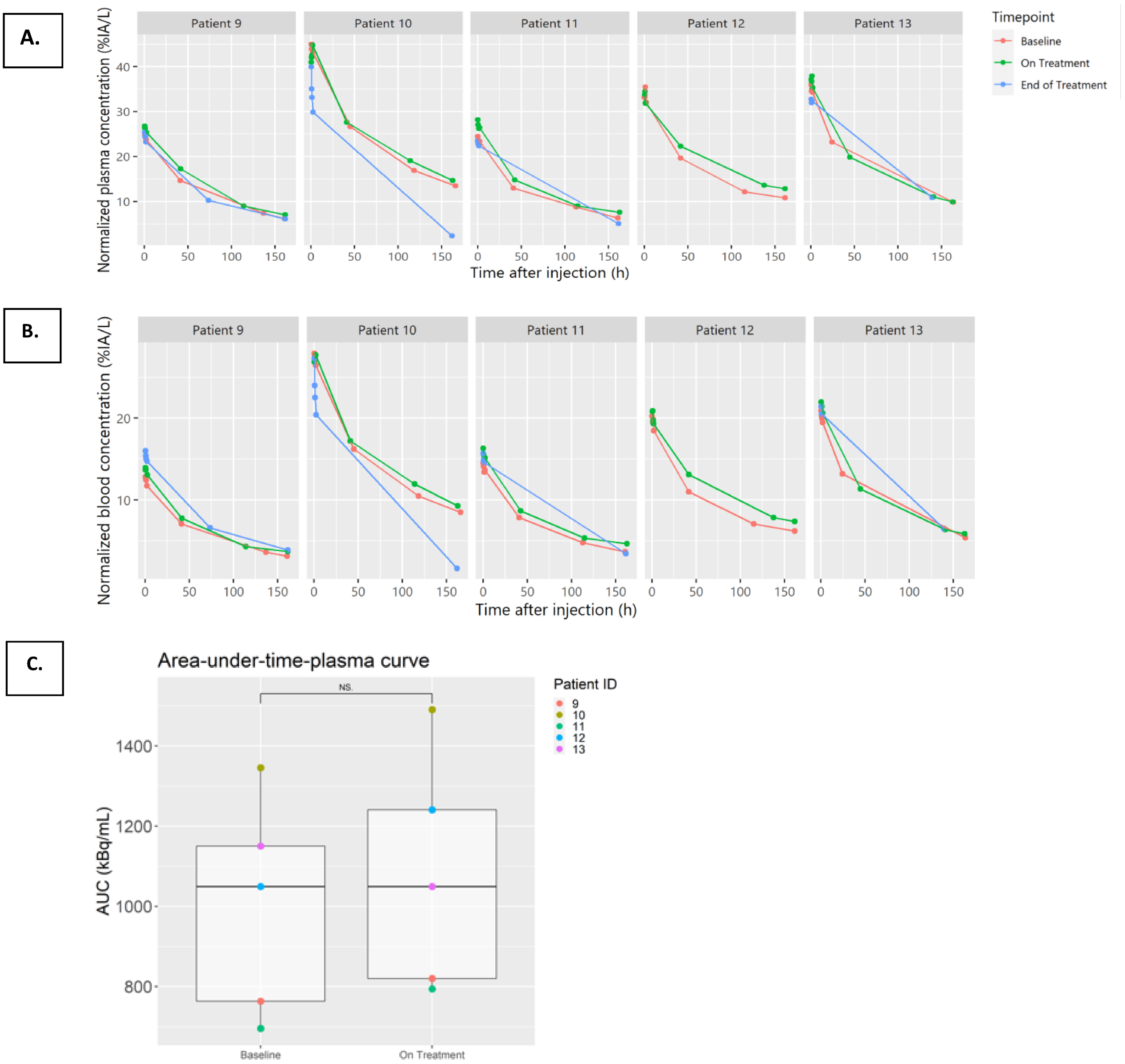

Supplemental Figure 3; *[^{89}Zr]Zr-durvalumab pharmacokinetics for the 22.5 mg dose cohort, based on measured activity concentration in venous blood samples.* ***(A)*** *Although absolute plasma concentrations between patients vary with a maximal plasma concentration straight after injection for patient 10, the within patient variability is low, with patient 10 being an outlier showing fast decrease after the end of treatment injection (PET data not included in analysis). The plasma concentrations are normalized for injected activity (37±1 MBq). Note that the curves at end of treatment show an overestimation due to limited data points included.* ***(B)*** *The blood concentrations*

follow the same trend as plasma concentrations. ***(C)*** *The mean total plasma supply expressed as area under time-activity concentration curve (AUC) does not differ between baseline and on-treatment.*

Abbreviations: *%IA/L = percentage measured activity in plasma or blood of total injected activity per liter plasma or blood. AUC = area under time-activity concentration curve.*

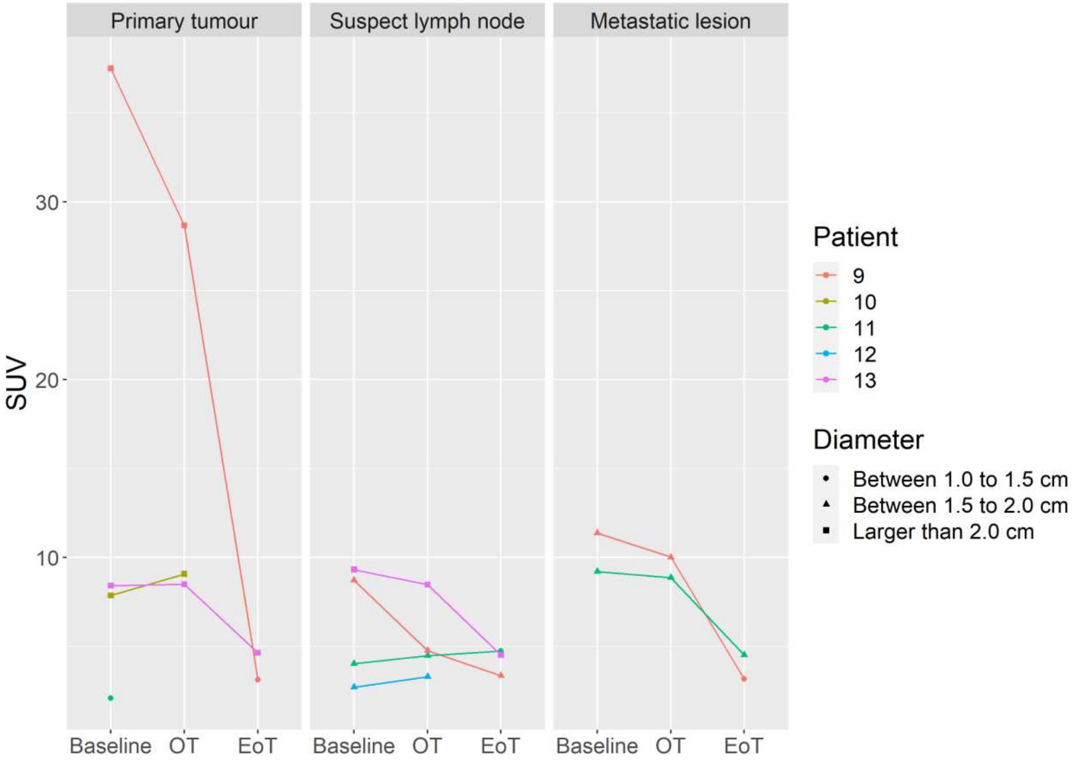

Supplemental Figure 4; *[^{89}Zr]Zr-durvalumab tumor uptake per lesion during treatment for patients in the 22.5 mg cohort in SUV_{peak}. A decrease on-treatment in the primary tumor and metastatic lymph node of patient 9, the metastatic lymph node of patient 13 and both (non-irradiated) metastatic lesions (patient 9 and 11) is shown. An increase in SUV_{peak} can be observed in the primary tumor of patient 10 and the metastatic lymph nodes of patient 11 and 12.*
